# The Use of Cyllin in the Treatment of Mediterranean Fever

**Published:** 1906-10-20

**Authors:** D. J. McNabb


### Recent Therapeutic Methods.

### THE USE OF CYLLIN IN THE TREATMENT OF MEDITERRANEAN FEVER.

By Fleet-Surgeon D. J. McNabb, R.N.

The following account in the Navy Blue Book of the treatment of 42 cases of Mediterranean fever will be of interest to general practitioners in seaboard towns and in remote districts from which recruits are drafted and to which invalids find their way during their period of recovery and convalescence:—

One man arrived in England in H.M.S. *Implacable*, which had come home to pay off. Some little time before leaving the Mediterranean he had been operated on for radical cure of inguinal hernia in Bighi Hospital. The operation was successful, and he was discharged cured, but on the passage home he contracted Mediterranean fever. In the 42 cases 2 deaths occurred, but as one was from hepatic abscess and one from pyæmia, they cannot be directly attributed to the fever. With the one exception, quoted above, all the cases occurred in patients who had been previously under treatment in the Mediterranean, and who had recovered sufficiently to be able to come home. In the general management of these cases it was found that the patients assimilated ordinary solid food with benefit to themselves, irrespective of high temperature. This is merely in accordance with the recorded experience of others. In some cases there was anorexia, which was probably brought about by the acute pain of the local symptoms.

Numerous drugs were tried without any appreciable result, the only one appearing to be of benefit being cyllin. This drug is a preparation of the Jeyes' Sanitary Compounds Company, and appears to be a derivative of creosote. The preparation used was the "pure cyllin," in palatinoids, each palatinoid containing 3 minims of the drug. The course of treatment by cyllin was preceded by a purgative such as calomel, and the drug was given at the rate of two palatinoids thrice daily. It is non-poisonous, and its administration was followed by no unpleasant results.

A table here is given showing an analysis of the cases, from which it will be seen that of the 42 cases under consideration 10 had neither pyrexia nor symptoms. These were dismissed from the list, and the remaining 32 dealt with. These 32 cases are divided into 24 which were *not treated with cyllin*, and 8 cases in *which the drug was tried*.

The average duration of the cases not treated worked out at 70.6 days, while that of the cases where the drug was used was 38.5 days.

### The Course of the Cases Indicated by the Temperature Chart.

M. H.—This was the longest case of any treated by this drug. Some general diminution in the range of the temperature is noticeable, with an occasional slight rise coincident with a severe attack of "neuritis" in the right arm. But from the commencement of the use of the drug the undulant character of the pyrexia disappeared.

P. S.—Temperature fairly high on admission, but fell on the fourth day of the cyllin treatment. After this it only rose to 101° on the day the drug was stopped, falling afterwards, the further fall being apparently hastened by the resumption of the cyllin.

L. A.—The administration of the drug produced a speedy fall, which was permanent.

T. G.—In this case the undulations of temperature were apparently interrupted by the drug. Immediately on its cessation there was an outburst of pyrexia, which seemed to be the last flicker, as there was no further trouble.

T. V.—In this case cyllin proved to be useless. High temperature was persistent, and the patient was markedly anæmic, the count of red blood-cells giving a little over 2,000,000 per c.mm. The chart goes to show the failure of the cyllin. The patient had been kept in bed while the treatment was carried out, and was given a nutritious diet; but my recollection is clear that, without using the cyllin, and without making any other change, his temperature fell markedly on allowing him to get up out of bed, and became normal after a couple of days in the open air.

P. C.—What promised to be a wave of pyrexia appears to have been cut short, and a normal temperature maintained as the result of the cyllin. In this case the "neuritis" was very severe, and persisted regardless of the temperature.

J. F.—A sudden fall of temperature was produced, followed by a slight rise and a second fall. Once this fall was established, it would appear that the cyllin had some anti-pyretic effect, as the accidental omission of the drug on February 14 caused an immediate, if slight, rise of temperature, which disappeared on resuming the drug. The same effect was produced from February 19 to 26.

H. C.—This case shows more clearly than any other the abrupt fall during a wave of pyrexia after six days' treatment with cyllin.

Of the 32 cases dealt with in these notes 21 suffered from various painful local affections (so-called neuritis). Of these 21 cases the region of the sacro-iliac joint was the seat of pain in 7 cases (5 right and 2 left). Three cases affected the trunk of the great sciatic nerve. The remaining 11 cases had the local symptoms in various nerves, muscles, and joints, other than the above mentioned, and none in anything like the same proportion. One case of sacro-iliac pain resulted in the formation of an abscess which pointed at the great sacro-sciatic notch, and I can recall another case from amongst those quoted here where a large lumbar abscess was discovered and dealt with. That the sacro-iliac affection is a joint disease appears to me clear, (1) from the excessive tenderness over the posterior aspect of the joint, and (2) that pressure on the crests of the iliac bones produced intense pain in the affected joint. I know that others better equipped than myself are making strenuous efforts to grapple with this disease, and all I wish to do is to suggest the value of intestinal disinfection in the effort to get the disease under control and to prevent the waste of tissue from persistent pyrexia. I also wish to draw attention to the preference for particular localities which is manifested by the local symptoms. Of the 42 cases 32 had pyrexia, and the average duration of illness was 65.3 days. The 8 cases treated with cyllin lasted 38.5 days, whereas those not treated with cyllin lasted 70.6 days.

